# Athletic equipment microbiota are shaped by interactions with human skin

**DOI:** 10.1186/s40168-015-0088-3

**Published:** 2015-06-19

**Authors:** Mariah Wood, Sean M. Gibbons, Simon Lax, Tifani W. Eshoo-Anton, Sarah M. Owens, Suzanne Kennedy, Jack A. Gilbert, Jarrad T. Hampton-Marcell

**Affiliations:** Northwestern University, Evanston, IL USA; Biosciences Division, Argonne National Laboratory, Lemont, IL USA; Graduate Program in Biophysical Sciences, University of Chicago, Chicago, IL USA; Department of Ecology and Evolutionary Biology, University of Chicago, Chicago, IL USA; Computation Institute, University of Chicago, Chicago, IL USA; MO BIO Laboratories Inc, Carlsbad, CA USA; Department of Surgery, University of Chicago, 5841 South Maryland Avenue, MC 5029, Chicago, IL 60637 USA; Marine Biological Laboratory, 7 MBL Street, Woods Hole, MA 02543 USA; College of Environmental and Resource Sciences, Zhejiang University, Hangzhou, 310058 China

**Keywords:** Gym microbiome, Athletic equipment, Microbiology, Niche communities, Next-generation sequencing

## Abstract

**Background:**

Americans spend the vast majority of their lives in built environments. Even traditionally outdoor pursuits, such as exercising, are often now performed indoors. Bacteria that colonize these indoor ecosystems are primarily derived from the human microbiome. The modes of human interaction with indoor surfaces and the physical conditions associated with each surface type determine the steady-state ecology of the microbial community.

**Results:**

Bacterial assemblages associated with different surfaces in three athletic facilities, including floors, mats, benches, free weights, and elliptical handles, were sampled every other hour (8 am to 6 pm) for 2 days. Surface and equipment type had a stronger influence on bacterial community composition than the facility in which they were housed. Surfaces that were primarily in contact with human skin exhibited highly dynamic bacterial community composition and non-random co-occurrence patterns, suggesting that different host microbiomes—shaped by selective forces—were being deposited on these surfaces through time. However, bacterial assemblages found on the floors and mats changed less over time, and species co-occurrence patterns appeared random, suggesting more neutral community assembly.

**Conclusions:**

These longitudinal patterns highlight the dramatic turnover of microbial communities on surfaces in regular contact with human skin. By uncovering these longitudinal patterns, this study promotes a better understanding of microbe-human interactions within the built environment.

**Electronic supplementary material:**

The online version of this article (doi:10.1186/s40168-015-0088-3) contains supplementary material, which is available to authorized users.

## Background

Indoor built environments (BEs) are the principal habitat of a large proportion of modern humans [1, 2]. Microbial communities are found on many BE surfaces, yet we know very little about how these communities form [3]. Understanding the microbiome of indoor surfaces will help us to design better buildings, where surface-mediated human-microbe interactions are optimized for health and safety [4].

From an evolutionary perspective, many BE surfaces are unfamiliar to microbes. BE habitats contain numerous chemically inert materials (plastics, paints, varnishes, metal alloys, etc.) that drive the assembly of BE-specific communities [4], which are mainly sourced from the human microbiome [5, 6]. Initiatives such as the Home Microbiome Project (www.homemicrobiome.com) have shown that individual homes have different microbial fingerprints that mirror the microbiota of their inhabitants [5]. However, the degree to which homes were found to be distinct varied depending on the surface type examined and how inhabitants interacted the surfaces. For example, when comparing across home environments, floors were the most distinct surfaces, while surfaces that interacted directly with human skin were the most similar [5].

Here, we investigate the microbiota of athletic facility surfaces. Previous studies of athletic facilities have focused almost entirely on the suspected presence of pathogens [7, 8], using traditional culturing methods that do not capture the broad taxonomic diversity present in the environment [9]. Previously, we have demonstrated that bacterial assemblages reach stable ecological states within hours on restroom surfaces and exhibit dynamic relationships with human occupants [10]. In this study, we completed hourly sampling of three different gyms across Chicago over 2 days in order to understand the forces governing microbial community assembly on different surfaces. We were particularly interested in how interactions with human skin influenced microbial ecosystem development and stability.

## Results and discussion

### Bacterial community structure was significantly different between equipment types

**Fig. 1:**
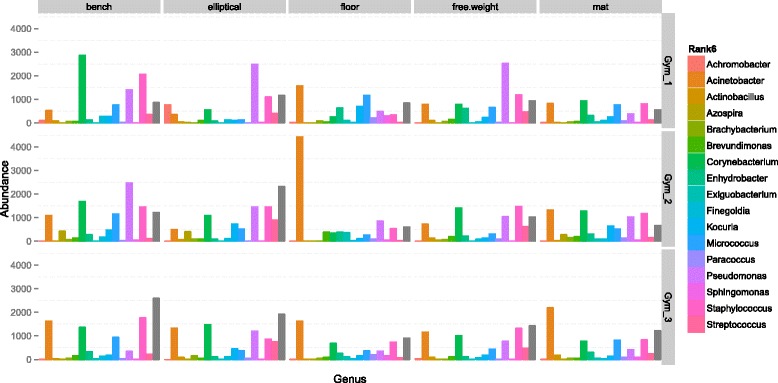
Equipment form distinct communities. Summary of bacterial community composition of abundant phyla associated with each surface at each athletic facility. To simplify community representation, OTUs less than 10 were discarded. While each surface displays a unique community structure, surfaces were similar across all facilities

**Fig. 2:**
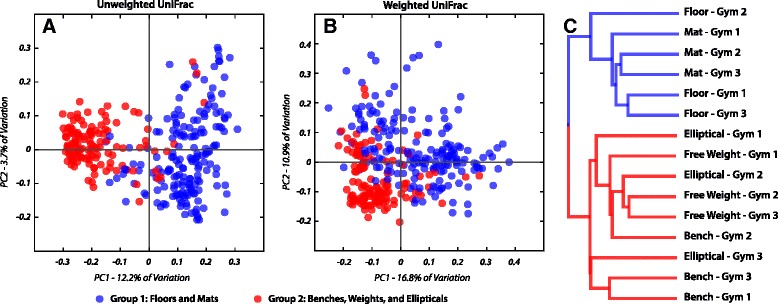
Interaction influences community diversity. Principle coordinate (PCoA) of unweighted (**a**) and weighted (**b**) UniFrac distances, and dendrogram of weighted UniFrac distances (**c**) between samples based on group identity. Group 1 (*blue*) consists of floors and mats; group 2 (*red*) consists of elliptical handles, free weights and benches

**Fig. 3:**
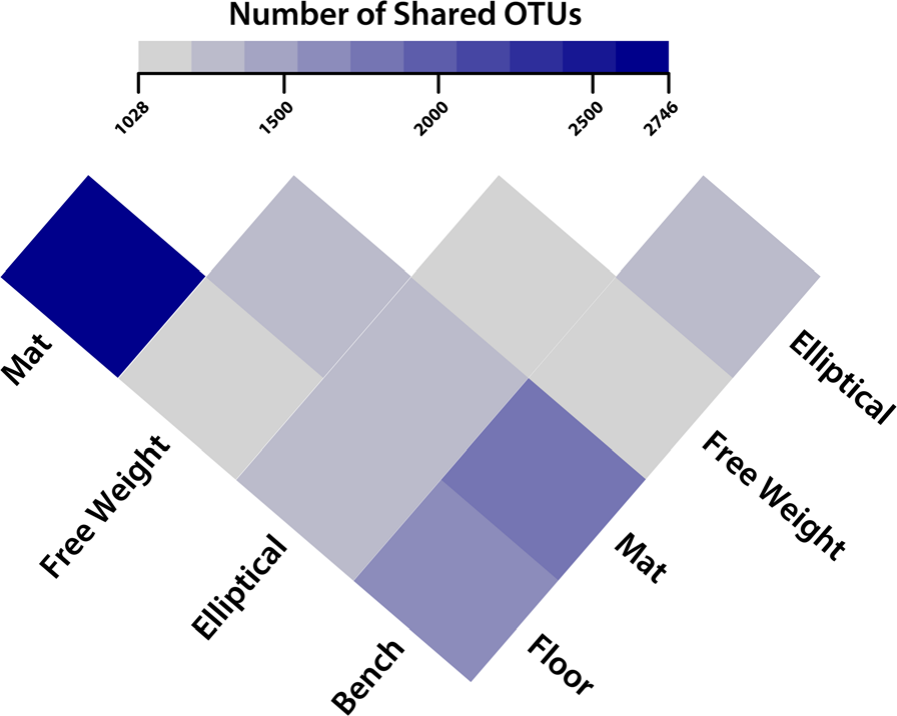
Bacterial sharing is related to mode of interaction. A heatmap displays shared OTUs between surface pairs (*mats*, *floors*, *ellipticals*, *free weights*, and *benches*). The quantity of shared OTUs is *colored* by gradient with increased OTUs represented by *dark purple*

**Fig. 4:**
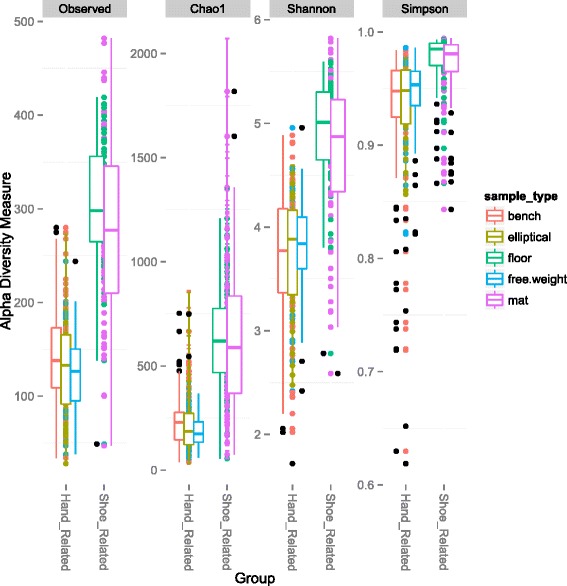
Equipment display novel communities. Summary of alpha diversity metrics of richness and evenness associated with each sample and its respective grouping. Observed species and Chao1 were plotted to measure richness. Shannon and Simpson indexes measured evenness

A total of 10,997,197 sequences were generated from 356 samples. These comprised a total of 50,293 non-singleton OTUs (Operational Taxonomic Units; 97 % identity) across all samples. Samples were normalized to a rarefaction depth of 800 sequences per sample. *Pseudomonas* and *Acinetobacter* were the most abundant genera across samples, comprising 9.3 and 8.6 % of all sequence reads, respectively (Fig. 1), while *Staphylococcus* (mean abundance 8 %), *Corynebacterium* (8 %), and *Micrococcus* (4 %) were also abundant. Bacterial community composition, pooled by facility, was significantly but weakly different between facilities (analysis of similarities (ANOSIM) *R* = 0.07; *p* < 0.001). However, it was significantly more different between surface types (ANOSIM: *R* = 0.3903; *p* < 0.001). When surfaces were grouped by their dominant mode of human interaction, i.e., contact with shoes (floors and mats) or hands (elliptical handles, free weights, and benches), the surfaces showed significantly distinct microbial community composition and structure (ANOSIM *R* = 0.5106; *p* < 0.001) and significant clustering (Fig. 2; Additional file 1: Figure S1). ‘Shoe’ surfaces harbored a larger core microbiome (Table 1) (microbes shared across 90 % of samples) than ‘hand’ surfaces (Fig. 3), and shoe surfaces displayed consistently higher richness and evenness than hand surfaces (Fig. 4).

**Table 1:** Bacterial genera which were considered core (found in all samples) to each surface type

Group 1			Group 2	
Floor	Mat	Bench	Weight	Elliptical
Staphylococcus	Staphylococcus	Staphylococcus	Staphylococcus	Staphylococcus
Pseudomonas	Pseudomonas	Pseudomonas	Pseudomonas	Pseudomonas
Micrococcus	Micrococcus	Micrococcus	Micrococcus	
Corynebacterium	Corynebacterium	Corynebacterium	Corynebacterium	
Kocuria	Kocuria		Streptococcus	
Enhydrobacter	Enhydrobacter			
Acinetobacter	Acinetobacter			
Rothia				
Paracoccus				
Exiguobacterium

OTUs closely related to the genus *Paracoccus* were significantly enriched on shoe surfaces (>1 % relative abundance; *p* value <0.001). The genera *Anaerococcus* (associated with vaginal samples [11]) and *Finegoldia* (associated with GI tract samples [12]) were most abundant (1.3, 1.2 %) on benches and were more abundant on free weights and elliptical handles (0.7, 0.5 %) than on mats and floors (0.4, 0.1 %), indicating that they are more closely associated with surfaces that frequently come into contact with human skin. *Bacteroides*, an abundant genus in the human intestinal tract, was more prevalent on elliptical surfaces (1.8 %) than on shoe surfaces. One genus that is associated with both groupings, *Enhydrobacter*, was most abundant on floors and free weights (3, 2.3 %). This genus is abundant on female skin and is generally associated with the buttocks [13].

We investigated the likely source environments for microbes detected on gym surfaces using SourceTracker trained on the Earth Microbiome Project database, which contains samples from host-associated and outdoor environments (Additional file 2: Figure S2). We found that bacterial taxa associated with the equipment surfaces across all three facilities were most likely to be sourced from human skin. These results are similar to what was found recently for restroom surfaces [10].

### Floor microbial communities are more stable than skin-associated communities

**Fig. 5:**
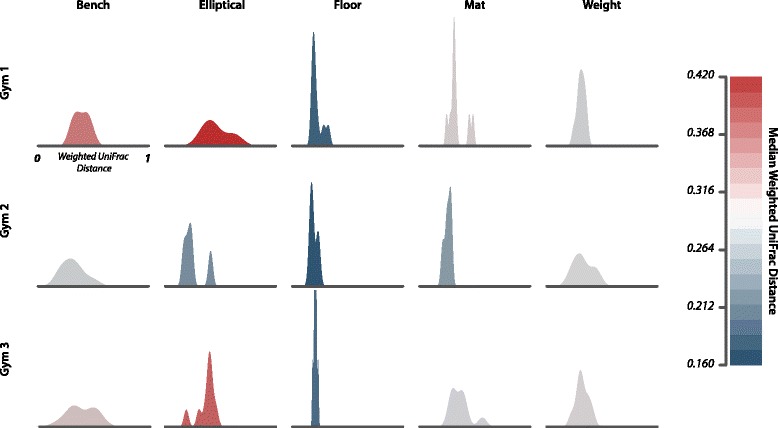
Community stability is equipment specific. Density plots displaying the stability of equipment-type samples in aggregate. *Floors*, *mats*, and *weights* show higher stability due to the narrow range of UniFrac distance, *floors* being the most stable. *Benches* and *ellipticals* display greater variation and volatility

The floor was found to have the most stable microbial community structure, with the lowest median UniFrac distance between floor samples, and a narrow distribution of these distances (Fig. 5). Conversely, bench and elliptical median UniFrac distances were larger, and the distributions were wider, suggesting that the beta-diversity variance was significantly greater for surfaces frequently in contact with skin (Fig. 5; permutational analysis of multivariate dispersions (PERMDISP), *p* < 0.001).

Despite the wide distribution of beta-diversity distances associated with bench and elliptical handle microbiota, OTU co-occurrence structure was non-random (*C* score, *p* values = 0.03 and 0.01, respectively). Non-random co-occurrence suggests that niche-based, non-random processes such as environmental filtering or competition among species are the dominant forces shaping community assembly [14]. The higher variance in beta-diversity distances within hand surface samples suggests turnover of distinct communities (sub-clusters), which represent multiple hosts, during the course of the day. Thus, deterministic forces seem to indirectly govern the microbial dynamics of bench and elliptical handle surfaces because the communities residing on these surfaces are derived from the skin microbiome. Conversely, the less volatile floor and mat communities did not display significant co-occurrence structure, which suggests that community assembly was governed by neutral forces [15].

We performed a meta-analysis to compare bacterial communities across two distinct built environments: public restrooms [10] versus our recreational facilities. In order to make the two data sets intercomparable, we analyzed the closed-reference OTU tables (OTUs that map to the Greengenes database). The restroom study served as good comparison for hand/skin- (i.e., soap dispenser and toilet seat) and shoe-associated (i.e., bathroom floor) samples. An alpha diversity analysis showed similar trends across studies, where shoe-associated samples display greater richness and evenness relative to skin-associated samples (Additional file 3: Figure S3). When samples were grouped by study (Additional file 4: Figure S4), surfaces displayed significant clustering (ANOSIM *R* = 0.489; *p* < 0.001) suggesting distinct bacterial assemblages based on surface type. The second ordination axis in Additional file 3: Figure S3 separated skin- and shoe-associated samples (upper and lower regions of the plot, respectively), independent of study.

**Fig. 6:**
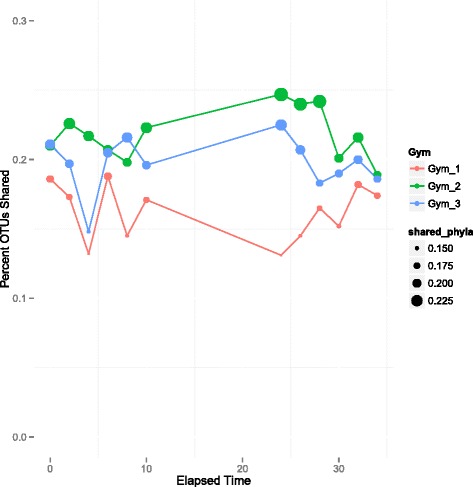
Shared phyla are not time dependent. Average shared phylotypes over time

The mean displacement of community composition through time was investigated by running distance comparison analyses, in which the UniFrac distance of samples, broken out by facility and equipment type, were measured against community composition at T-0. The fraction of shared phylotypes within each facility at each time point was also measured. Distance comparison analysis showed no significant differences across time (Student’s *t* test; *p* > 0.05). Shared phylotype analysis revealed that 13–19 % of phylotypes in gym 1 were shared with the initial time point, gym 2 maintained between 19 and 25 % shared phylotypes, and gym 3 maintained between 14–23 % shared OTUs. It did not, however, reveal any directional shift in microbial community overlap over 2 days of sampling (Fig. 6). These results suggest that microbial communities in athletic facilities fluctuate around a common centroid, as might be expected in a building with routine human occupancy characteristics. Thus, the passage of time did not lead to any discernible trends in microbial community structure, as further supported by the largely insignificant correlation between UniFrac distance and time (Mantel, gyms 1 and 2: *p* > 0.05, gym 3: *p* = 0.05).

Much remains unknown about the way microbial communities colonize and persist in indoor environments. Athletic facilities, which contain many BE sub-niches (surface types), present an ideal opportunity to study the forces shaping microbial community structure and dynamics in a BE setting. The results of this study suggest that microbial community structure is primarily defined by the mode of human interaction with a surface and not the geographic location of an athletic facility. This conclusion was demonstrated through the distinct clustering of shoe- and hand-associated surface communities, independent of gym location.

The results also suggest that increased interaction between inert surfaces and multiple distinct bacterial sources (e.g., human skin from different individuals) leads to an increase in community turnover. Indeed, surfaces that interacted with human skin were more likely to have a higher variance of UniFrac distances, suggesting a greater range of community profiles. This is in contrast to the findings of the Home Microbiome Project, in which human skin-associated communities experienced less community turnover than shoe-associated communities [5]. In the Home Microbiome Project, surfaces were exposed to the same individuals continually, whereas gym surfaces were exposed to different human hosts each time the equipment was used. Hence, human interaction can have the opposite effect on surfaces depending on the pattern of interaction. Co-occurrence analysis showed that despite the wide range of community structures found on hand surfaces (elliptical handles, free weights, and benches), the patterns of species co-occurrence were non-random. The human microbiome is shaped by ecological selection and the maintenance of distinct communities, resulting in non-random co-occurrence [16]. Therefore, the major factor driving beta-diversity variance for these hand surfaces was likely contact with human skin and potentially exacerbated by human sweat, a prevalent substance in an athletic facility. Given time, we would expect the selective signature (i.e., non-random co-occurrence) to dissipate in the absence of humans. BE surfaces are relatively inert and resource-poor, especially when compared to a human host, and it is unlikely that microbes are active for long in these environments (e.g., after moisture and oils from the skin are gone). This helps explain the differentiation between shoe- and hand-associated surfaces. Interestingly, skin-associated free-weight communities showed random co-occurrence patterns. This may be due to the fact that some metal surfaces, such as, in this case, the weight handle, have anti-microbial properties, which may prevent a host microbial signature from persisting as efficiently as on the other equipment types [17].

## Conclusions

In this study, we demonstrate that skin-associated surface communities are mutable and take on the fingerprint of the humans with which they come in contact. BE surfaces that do not interact with human skin exhibit stable community structure and lack detectable ecological selection, which fits with our model that most BE surfaces are inert, desert-like environments when compared to host systems [10].

This project is the initial investigation of an entire subsection of the built environment yet to be explored using high-throughput techniques and could provide the impetus for future studies on aspects of the athletic facility. Stemming from this research, a potential follow-up study could focus on the degree of persistence human microbial fingerprints leave on various gym surfaces. In such a study, human hosts could sample themselves as well as the surface with which they interacted. This would provide researchers with a baseline microbial signature for each human-microbe interaction, allowing for the examination of the resilience of the signature over time and how this resilience changes once another human host introduces a new signature to the equipment. Another follow-up issue worth investigating is how sweat might alter the skin microbiome and modify the dispersal/persistence of human-associated microbes on BE surfaces. Without isolating sweat as a factor of change, we cannot be certain if sweat will significantly drive dispersal and persistence patterns over a short interval [6]. Further studies could also investigate the impact of cleaning on microbial communities on gym equipment. For each equipment type, different cleaning materials made of different active ingredients could be administered after a particular microbe host used each piece, and again the equipment could be periodically sampled to examine the effects each cleaner has on that microbial community.

Humans drastically impact microbial niches in the built environment, whether through direct contact or through the manipulation of the physicochemical properties of BE surfaces. Understanding our own influence on the microbial BE will ultimately teach us how to build healthier spaces for ourselves and harness the power of our microscopic companions.

## Methods

Puritan sterile cotton swabs (model no. 25–806 1WC) were used to obtain samples from three different university recreational facilities in the greater Chicago area: gym 1, gym 2, and gym 3. Each facility was sampled over its own respective 2-day time series. Gyms 1 and 2 were sampled 4 days apart while gyms 2 and 3 were sampled 12 days apart. Samples were collected every other hour on 2 weekdays beginning at 8:00 am and ending at 6:00 pm each day. At each athletic facility, five different sites were sampled: elliptical machines (left handle), exercise mats (entire surface), weights (handles), weight benches (entire human-contacted surface), and the floor (near entryway). Two samples were taken from two separate surfaces of each site at each time point, with the same exact spots on each site being sampled every time. Each site was swabbed for between 3 and 15 s. At each sampling time point, a total of 10 samples were taken (duplicate samples from five surfaces), with a total of 60 samples per day and 120 samples over the 2-day sampling period. Each gym yielded 120 samples, except gym 1, where four samples at 6:00 pm on day 2 could not be obtained. A total of 356 samples were collected for processing and analysis. Descriptive metadata was collected for each sample, including the date, time, location, temperature and humidity of the entire facility at each time point, equipment make/model, and material type of each sample, cleaning regimen and reagents used, and number of patrons at the athletic facility for that day. Samples were transported on ice to Argonne National Laboratory (within 24 h) and stored at −80 °C.

All samples were prepared using a modified version of the Mo BIO UltraClean®-htp 96 Well Swab DNA Kit (MO BIO). Samples were purified using the Zymo ZR-96 DNA Cleanup and Concentrator™-5 kit according to Zymo Protocol (Zymo). DNA was prepared for PCR using the protocol from the swab kit. Fragments were amplified in a 26-uL reaction, using 12 uL PCR-grade H_2_0, 10 uL 5 mM HotMasterMix (5 Prime Inc), 1 uL forward primer, 1 uL 5 mM reverse primer, and 2 uL DNA. PCR was performed following the conditions outlined in Caporaso et al. [18]. During PCR, the highly variable V4 region of the 16S rRNA gene was amplified using region-specific primers and labeled with a unique 12-base Golay barcode sequence contained in the reverse primer of each sample [19]. DNA amplicons were quantified utilizing a plate reader. After quantification, distinct volumes from each well were pooled in order to ensure each amplicon was represented equally. Pools were quantified using the Qubit (Invitrogen). Sequencing of the prepared library was performed on the Illumina MiSeq platform, using the sequencing primers and procedures described in the supplementary methods of Caporaso et al. [19].

Using the Quantitative Insights into Microbial Ecology (QIIME) 1.7.0 toolkit, 13,664,316 sequences were processed [18]. Barcoded samples were de-multiplexed. Open-reference OTU picking using the May 2013 Greengenes release was performed, generating 50,293 non-singleton OTUs at 97 % nucleotide identity. The sequences were clustered at 97 % identity with the Greengenes database [19]. Reads that did not match a reference sequence were then clustered de novo at 97 % identity. Representative sequences were aligned using PyNAST [20], and those that failed to align were discarded. These representative sequences were used to assign taxonomy to each OTU cluster using the RDP classifier [21]. A phylogenetic tree was built using FastTree 2.0, which was then used to calculate UniFrac distances [14].

The samples were first analyzed using principle coordinate analysis [22] plots and the OTU network built using Cytoscape. Beta-diversity clustering was analyzed using ANOSIM for categorical variables and Mantel tests for continuous variables (e.g., time). Analysis of variance (ANOVA) was run to assess significant differences in relative abundance of OTUs on different surface types. A PERMDISP test was run to assess the significance of beta-diversity distribution variation between sample types. Core microbiomes, SourceTracker [23], and shared phylotypes were computed using QIIME 1.7.0. The co-occurrence analyses were carried out by calculating checkerboard scores and comparing these to a null-model using the oecosimu script as described in Barberán et al. [14] from the vegan package, processed in RStudio™.

### Availability of supporting data

All sequence data will be made available through FigShare, http://dx.doi.org/10.6084/m9.figshare.1320867.

## Additional files

Additional file 1: Figure S1. OTU networks display community interaction. To reduce network complexity, the OTU table was filtered to include only OTUs comprising greater than 50 reads and to remove OTUs detected only in a single sample. Network input files were constructed using QIIME’s make_otu_network.py script and were visualized in Cytoscape using the edge-weighted, spring-embedded layout. Additional file 2: Figure S2. Human skin is a dominant source across communities. SourceTracker models were generated on equipment surfaces for recreational facility. Sourced environments were taken from the Earth Microbiome Project database. Point size represents predicted source contribution to each surface. Additional file 3: Figure S3. Variation between environments are host-associated. Summary of alpha diversity metrics of richness and evenness associated with two different built environments (restroom and gym). Samples were grouped based likely on interaction, human skin (hand) versus inert surface (shoes). Observed species and Chao1 were plotted to measure richness. Shannon and Simpson indexes measured evenness. Additional file 4: Figure S4. Built-environments display distinct communities. Principle coordinate (PCoA) of weighted UniFrac distances, between samples associated with bathroom and gym surfaces. Bathroom surfaces were taken from the Earth Microbiome Project database and compared to gym surfaces using closed reference OTU selection.
