# Supplementary figures and images for: Athletic equipment microbiota are shaped by interactions with human skin
Source: Microbiome. 2015 Jun 19;3:25. doi: 10.1186/s40168-015-0088-3 (PMC4480904; doi:10.1186/s40168-015-0088-3)

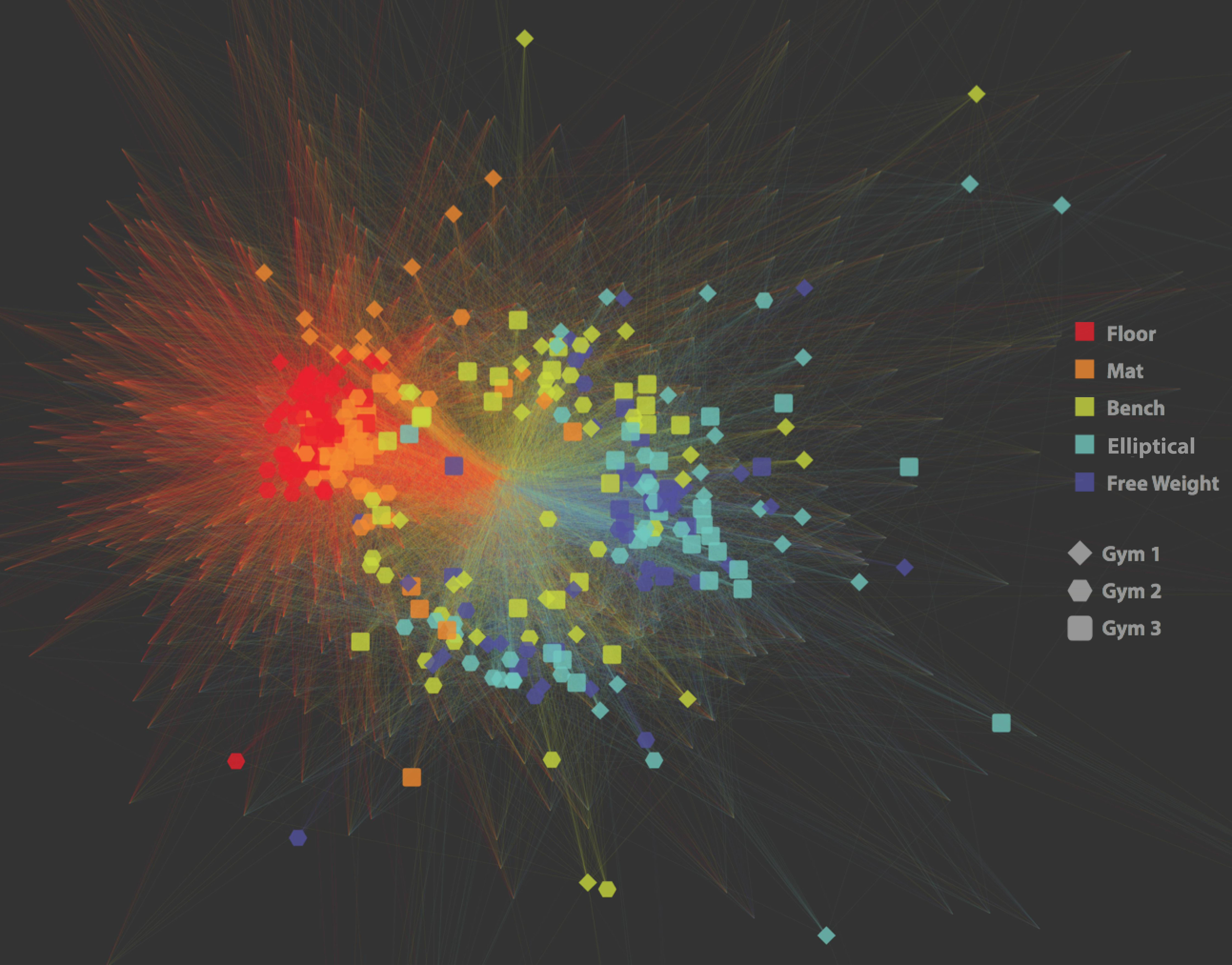

Supplement: Additional file 1: Figure S1. — OTU networks display community interaction. To reduce network complexity, the OTU table was filtered to include only OTUs comprising greater than 50 reads and to remove OTUs detected only in a single sample. Network input files were constructed using QIIME’s make_otu_network.py script and were visualized in Cytoscape using the edge-weighted, spring-embedded layout. [file 40168_2015_88_MOESM1_ESM.png]

Source

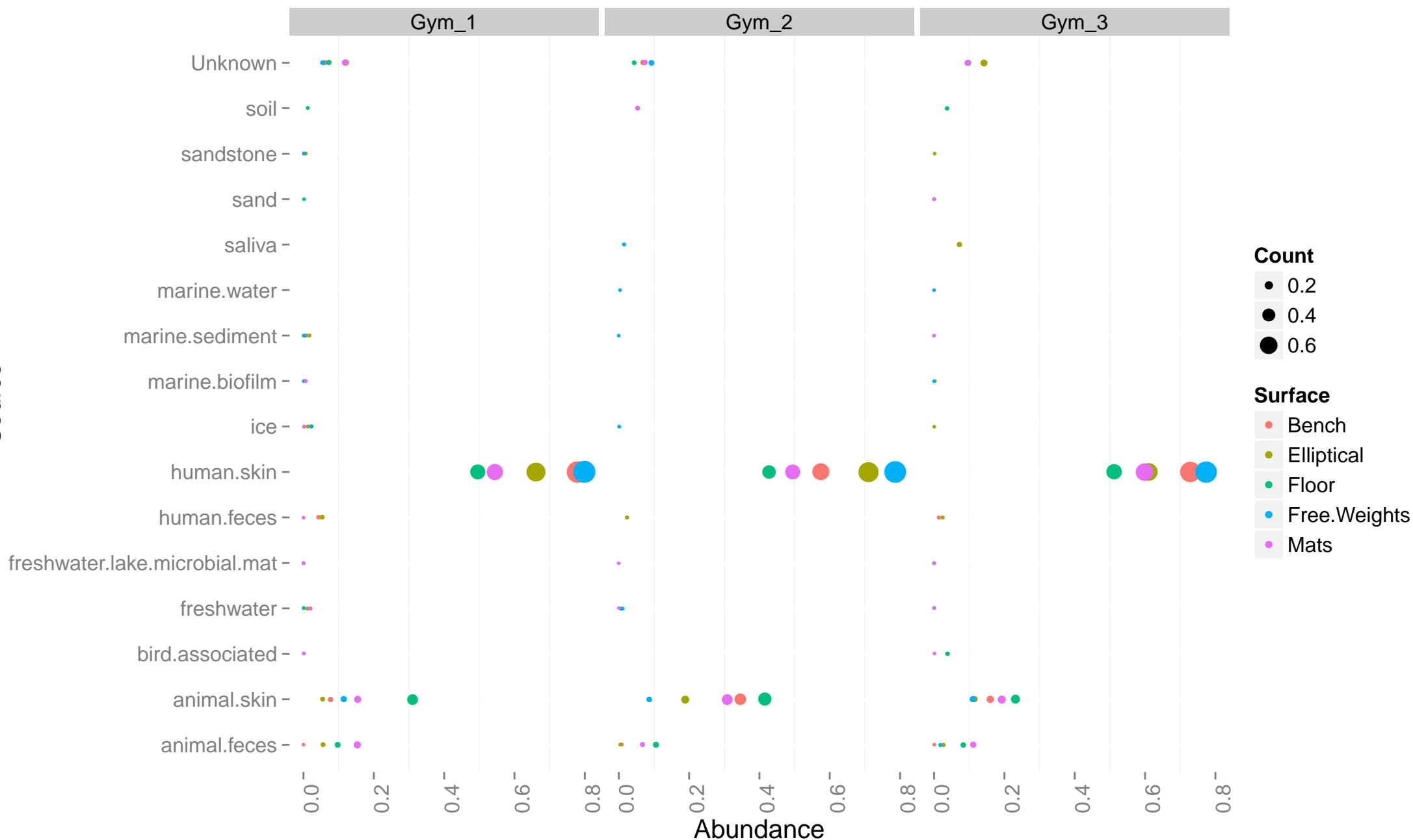

Supplement: Additional file 2: Figure S2. — Human skin is a dominant source across communities. SourceTracker models were generated on equipment surfaces for recreational facility. Sourced environments were taken from the Earth Microbiome Project database. Point size represents predicted source contribution to each surface. [file 40168_2015_88_MOESM2_ESM.pdf]

Alpha Diversity Measure

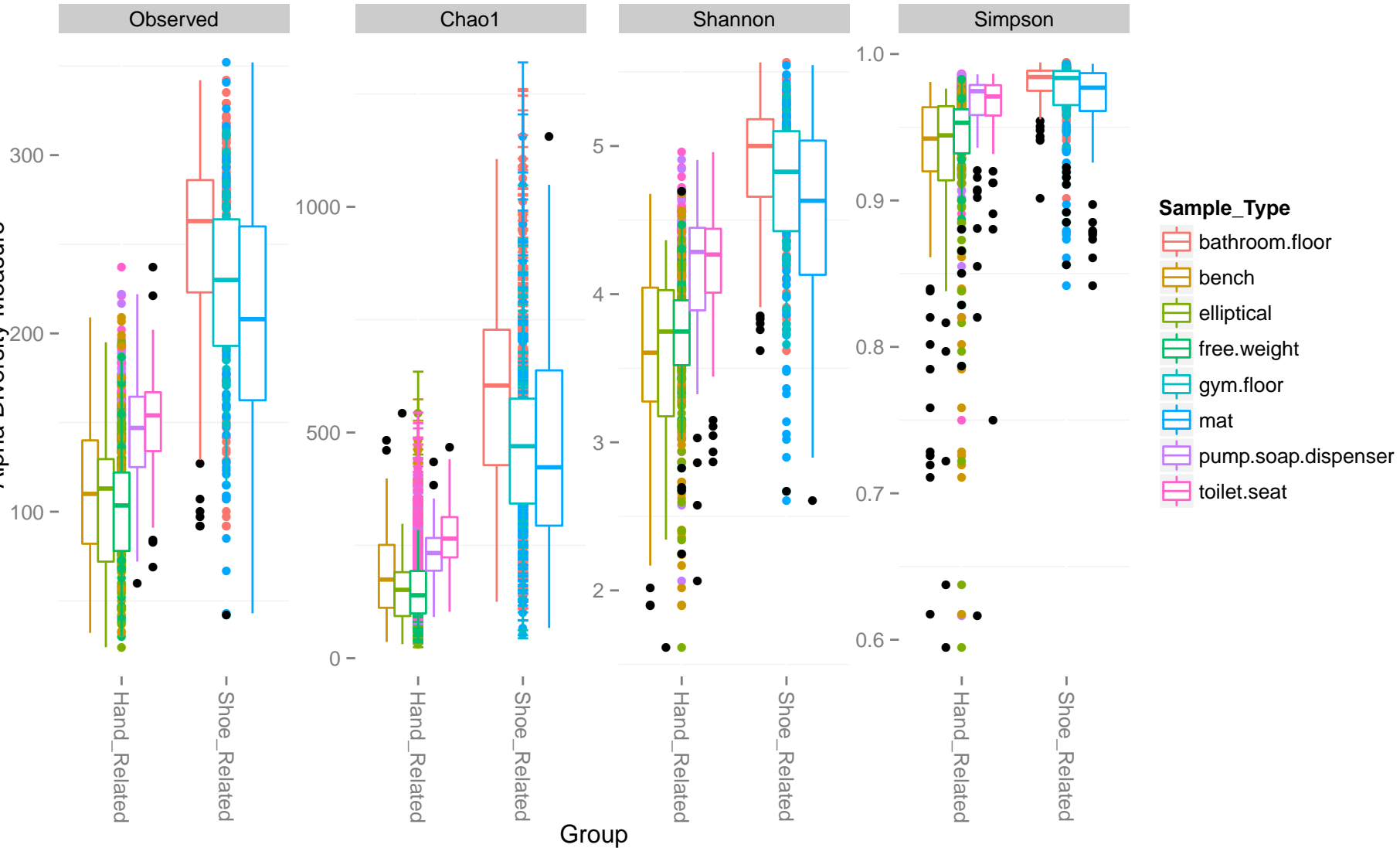

Supplement: Additional file 3: Figure S3. — Variation between environments are host-associated. Summary of alpha diversity metrics of richness and evenness associated with two different built environments (restroom and gym). Samples were grouped based likely on interaction, human skin (hand) versus inert surface (shoes). Observed species and Chao1 were plotted to measure richness. Shannon and Simpson indexes measured evenness. [file 40168_2015_88_MOESM3_ESM.pdf]

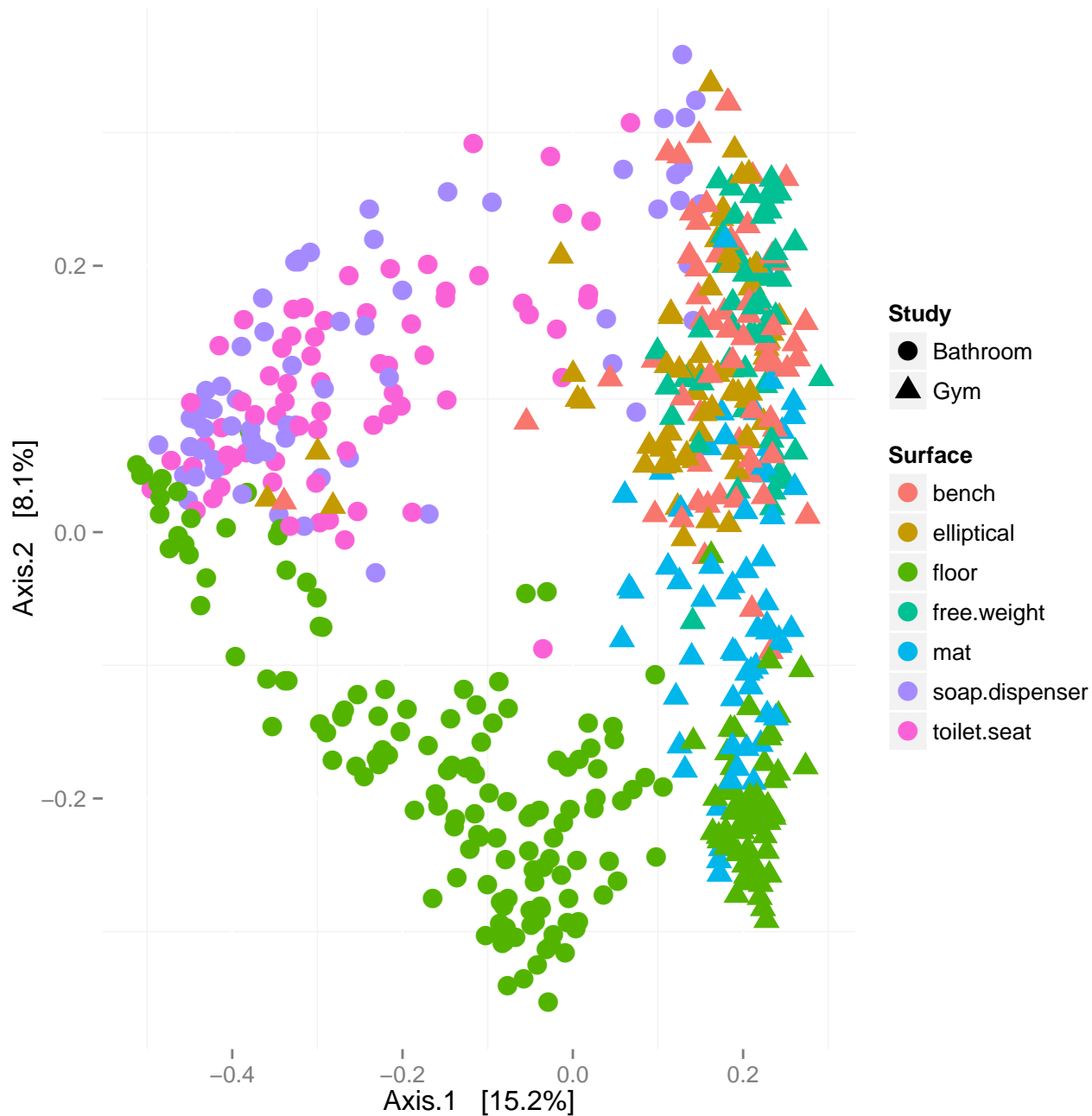

Supplement: Additional file 4: Figure S4. — Built-environments display distinct communities. Principle coordinate (PCoA) of weighted UniFrac distances, between samples associated with bathroom and gym surfaces. Bathroom surfaces were taken from the Earth Microbiome Project database and compared to gym surfaces using closed reference OTU selection. [file 40168_2015_88_MOESM4_ESM.pdf]
